# Female Sexual Polymorphism and Fecundity Consequences of Male Mating Harassment in the Wild

**DOI:** 10.1371/journal.pone.0000580

**Published:** 2007-06-27

**Authors:** Thomas P. Gosden, Erik I. Svensson

**Affiliations:** Department of Animal Ecology, Lund University, Lund, Sweden; University of Texas Arlington, United States of America

## Abstract

Genetic and phenotypic variation in female response towards male mating attempts has been found in several laboratory studies, demonstrating sexually antagonistic co-evolution driven by mating costs on female fitness. Theoretical models suggest that the type and degree of genetic variation in female resistance could affect the evolutionary outcome of sexually antagonistic mating interactions, resulting in either rapid development of reproductive isolation and speciation or genetic clustering and female sexual polymorphisms. However, evidence for genetic variation of this kind in natural populations of non-model organisms is very limited. Likewise, we lack knowledge on female fecundity-consequences of matings and the degree of male mating harassment in natural settings. Here we present such data from natural populations of a colour polymorphic damselfly. Using a novel experimental technique of colour dusting males in the field, we show that heritable female colour morphs differ in their propensity to accept male mating attempts. These morphs also differ in their degree of resistance towards male mating attempts, the number of realized matings and in their fecundity-tolerance to matings and mating attempts. These results show that there may be genetic variation in both resistance and tolerance to male mating attempts (fitness consequences of matings) in natural populations, similar to the situation in plant-pathogen resistance systems. Male mating harassment could promote the maintenance of a sexual mating polymorphism in females, one of few empirical examples of sympatric genetic clusters maintained by sexual conflict.

## Introduction

Female fitness costs from mating conflicts with males are expected to drive sexually antagonistic coevolution [1] causing males and females to coevolve rapidly in a “chase-away” process [2]. Laboratory studies on model organisms such as *Drosophila* have revealed genetic and phenotypic variation in female response towards male mating attempts [3]–[7] and demonstrated sexually antagonistic co-evolution driven by mating costs on female fitness [3], [5], [8], [9]. Such mating conflicts have been suggested to increase reproductive isolation between populations [10] either in sympatry [11] or in allopatry [12] following secondary contact [13]. Rapid divergence of populations with differing levels of sexual conflict has been demonstrated in laboratory experiments on both fruit flies (*Drosophila melanogaster*)[3] and dung flies (*Sepsis cynipsea*)[14]. But a recent theoretical model found that sexually antagonistic mating interactions can lead to females forming different genetic clusters effectively preventing males from participating in a traditional coevolutionary chase away [2], with males instead become trapped between the clusters in a “buridan's ass” regime [11] (classical paradox; an ass placed between two equal piles of hay will starve as it will be unable to make any rational decision to start eating one rather than the other). Once such female morphs have been formed, ongoing and chronic sexual conflict should maintain the morphs through frequency-dependent selection favouring the rarer morphs [15], [16]. There is very little field data for these types of conflicts and most of the evidence for sexual mating conflict comes from laboratory experiments [3], [9], [17], [18]. Females could potentially respond to male mating harassment either by physically resisting male mating attempts [18]–[21], or by evolving fitness tolerance to the damage inflicted from extra matings [3], [6], [7], [22]. However, there is no data from natural populations on the consequences of differing female resistance/tolerance levels towards male mating attempts at the intraspecific level, similar to the resistance/tolerance dichotomy in plant/pathogen interactions [23].

**Figure 1:**
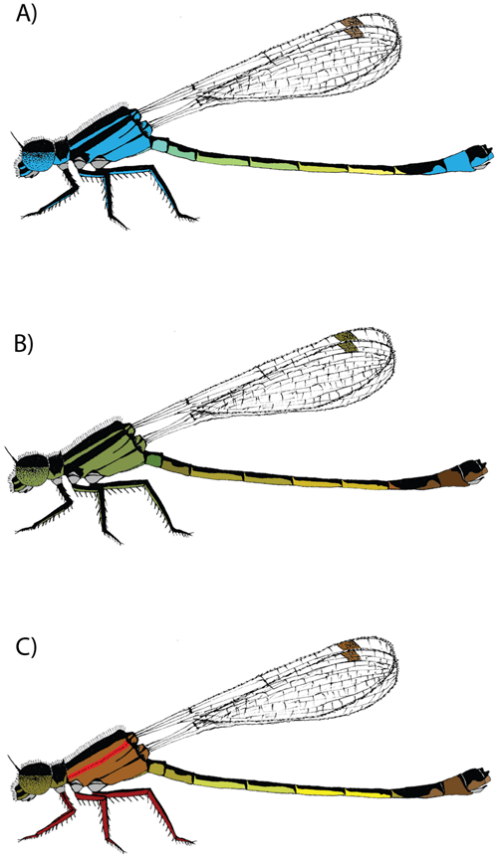
The three female morphs of *Ischnura elegans*. Morph is controlled by a single autosomal locus with three different alleles [25]. The six possible different genotypes are subject to a dominancy hierarchy where Androchrome (male mimic) (A) > Infuscans (B)> Infuscans obsoleta (C).

We performed experimental studies in natural populations of the colour polymorphic damselfly *Ischnura elegans* to examine sexual conflict over matings and associated fecundity effects on females. Females in this species occur in three discrete colour morphs, Androchromes, Infuscans and Infuscans-obsoleta (Fig 1). Of these three morphs, Androchrome females are considered to be “male mimics”, based on their blue colouration and melanic patterning which is identical to males [24]. Genetics of morph determination is due to a single autosomal locus with three different alleles in a dominance hierarchy and with sex-limited phenotypic expression in only females [25]. These three female morphs are thought to be maintained by frequency-dependent sexual conflict, in which common morphs suffer from excessive male mating harassment and apostatic selection, since males form search images directed towards common morphs [15], [26]. Although population genetic modelling and field data on fecundity-variation in the morphs indicate that frequency-dependent sexual conflict over mating maintains this polymorphism, direct evidence for male mating harassment has yet to be demonstrated. To obtain such data, we dusted males with one of five different fluorescent colours [27] on the clasping organ located at the end of the abdomen (cerci) and on their genitalia and released the dusted males in three field populations in southern Sweden. Using this experimental design, we were able estimate levels of resistance and mating rates for the three female morphs by looking for dust on the thorax, which indicates a male clasping attempt, and on the female's genitalia, which shows that mating had occurred with a dusted male. Females were subsequently set up in oviposition jars and left to lay eggs for two days before being released. We compared the overall mating rates of the morphs, morph-differences in resistance towards mating attempts and the effects of realized matings and mating attempts on female fecundity. Based on sexual conflict theory, we expected to find differences between the morphs in the number of matings received, their ability to reject male mating attempts and the fecundity-consequences of both mating attempts and realized matings.

## Results

**Figure 2:**
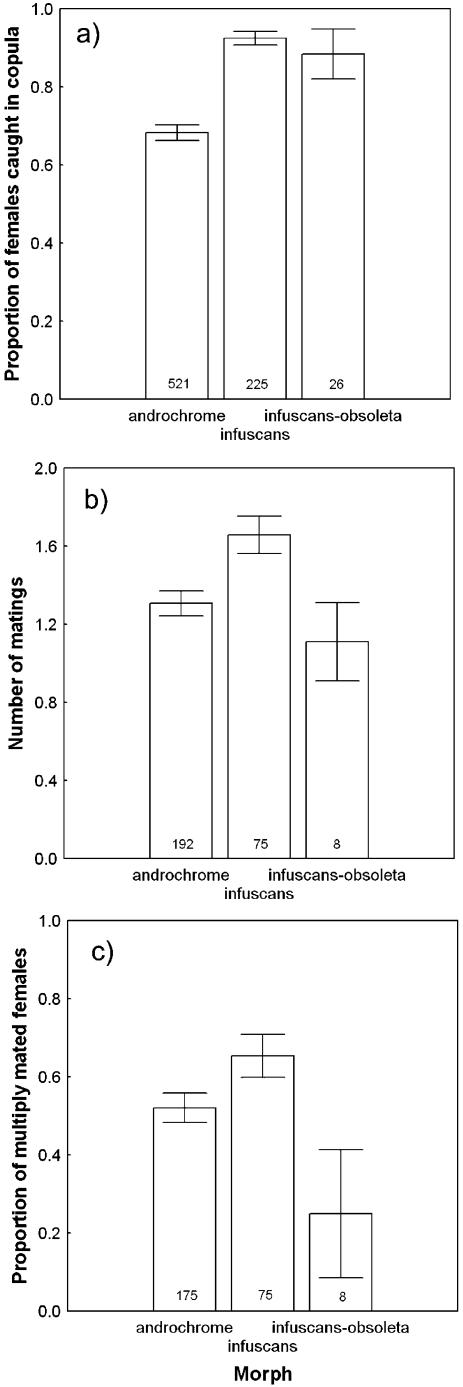
Differences in mating behaviour between three female colour morphs. a) Proportion of females caught in copula in the field ±SE (χ^2^ = 60.229, d.f. = 2, P = <0.001; N = 772). b) Average number of matings obtained by each morph ±SE (F_2,269_ = 4.014, P = 0.019; N = 275). Infuscans-obsoleta was not significantly different from either morph (Tukey HSD A = 0.6202, I = 0.1551) although the sample sizes for this morph were low (N = 8). c) Proportion of multiply mated females ±SE (χ^2^ = 6.981, d.f. = 2, P = 0.031; N = 258). All tests remain significant if the rarest morph, Infuscans-obsoleta, is removed from the analysis.

The proportion of field-caught females that were found in copula differed significantly between the three different morphs (Fig 2a). Androchromes had a significantly lower probability of being found in copula compared to the two other morphs. There were also significant differences in the number of matings obtained by the three morphs (Fig 2b), and androchromes obtained significantly fewer matings than infuscans females (Tukey HSD = 0.028, Fig 2b). Finally, the proportion of multiply mated females differed between the morphs (Fig 2c), confirming that infuscans females had a higher mating rate than the two other morphs.

**Figure 3:**
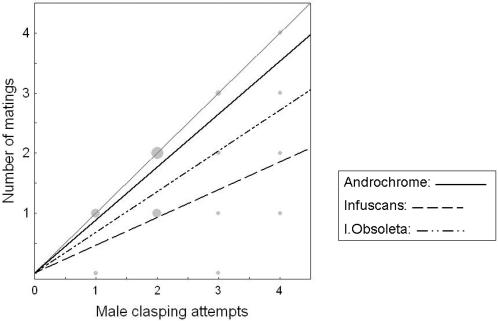
Morph-specific variation in female resistance to male mating attempts. Relationships between the number of matings (Y-axis) and the number of male clasping attempts received by each morph (X-axis). The fine dotted line marks a (hypothetical) 1∶1 slope, where every clasping attempt by a male ends in a mating. Regression lines below the 1∶1 relationship reveals morph-specific levels of female resistance towards mating attempts from males. The regression slopes differ significantly between the three female morphs (Morph * No. Male Claspings: F_2,266_ = 5.373, P = 0.005). Differences between androchrome females (solid regression line) and infuscans-females (dashed line) remains significant even if the morph with the lowest sample size (Infuscans obsoleta (dash-dot line)), is excluded from the data-set (F_1,260_ = 10.072, P = 0.002). Regression slopes of both androchrome females and infuscans-females differ significantly from unity (upper 95 % CL: 0.987 and 0.697 respectively), showing that both morphs do not accept all male mating attempts and show some degree of resistance. Sizes of the data points are proportional to the sample size at each position (range: 1–97 mean = 23.1).

Across all three morphs, the number of realized matings was significantly and positively related to the number of male clasping attempts (Fig 3: F_1,266_ = 19.637, P<0.001). The three morphs differed significantly in the relationship between the number of matings and the number of male clasping attempts (Fig 3). The overall positive relationship between realized matings and mating attempts indicates that morph-specific copulation frequencies in the field do, to some extent, reflect the total number of previous male mating attempts (Fig 3). Hence observed copulation rates in the field will partly reflect the true degree of male mating harassment, as we have previously argued [15].

The regression slopes of all three morphs differed from a 1∶1 slope, which is the relationship that would be expected in the absence of female resistance, i.e. if all male clasping attempts resulted in matings (Fig 3). Thus, all three female morphs showed some degree of resistance towards male mating attempts, although the magnitude of this resistance differed, resulting in morph-specific regression slopes (Fig 3: A; Y = 0.882x, I; Y = 0.463x, IO; Y = 0.679x). The slopes were significantly lower than 1 in both androchrome females (upper 95 % CL: 0.987, Fig 3; dark solid line) and in infuscans females (upper 95 % CL: 0.697, Fig 3; dashed line). This resistance-difference between these two common morphs (androchrome and infuscans) remained significant even when the rarest morph (infuscans-obsoleta) was excluded from the analysis (Fig 3).

**Figure 4:**
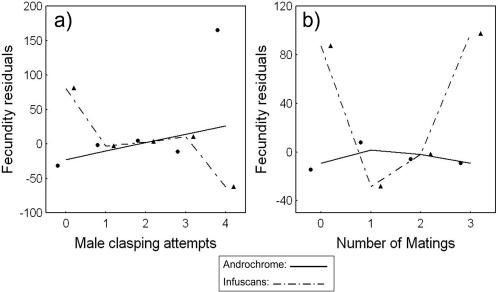
Fitness functions (cubic splines) showing morph-specific relationships between fecundity and minimum recorded number of mating attempts and realized matings. The fecundity-effects of population and year were removed prior to this analysis (fecundity-residuals are shown on Y-axis). Androchrome females (n = 200, solid lines and circles) and Infuscans-females (n = 78, broken lines and triangles), differ both with respect to a) Male clasping attempts (Morph * No. claspings: χ^2^ = 7.657, d.f. = 1, P = 0.006) and with respect to b) Number of matings (Morph * No. matings: χ^2^ = 4.755, d.f. = 1, P = 0.029). Interaction tests were estimated in generalized linear models (GLZ; with Poisson error, see methods). Because there were only 5 individuals across all morphs that had mated four times, these were excluded from the final model, however, results remained significant with their inclusion. Data-points show mean fecundity values for each particular morph and mating category.

There were significant differences between androchromes and infuscans females in how the number of male clasping attempts affected fecundity (Fig 4a) as well as how the number of obtained matings affected fecundity (Fig 4b). The data indicated that androchrome females were less sensitive than infuscans females, in terms of their fecundity, to both male mating attempts (claspings) and towards realized matings (Fig 4a,b). The relationship between fecundity and number of matings in infuscans females was U-shaped (Quadratic selection coefficient: s^2^ = 103.184 SE±42.936, F_1,72_ = 5.775, P = 0.019, N = 78) indicating that fecundity in this morph is maximized with either few or many matings.

## Discussion

Our results have revealed differences between morphs in both their resistance and their tolerance to male harassment and multiple mating from empirical field data. These morph-specific differences in resistance and tolerance to male mating harassment and realized matings are similar to resistance-tolerance variation in plant-pathogen interactions [23] where plants can cope with natural enemies by being either tolerant or resistant, or both depending on the associated costs. In the case of *I. elegans*, androchrome females show a more stable response to both increased harassment (Fig 4a) and increased matings (Fig 4b) and also mate less frequently than infuscans females (Fig 2), which could be due either to male mimicry making them more difficult for males to detect [28] or from active male mate choice for the more fecund infuscans females [24]. However, both tolerance and resistance towards male mating attempts and realized matings are likely to be costly, which is indicated by the fact that androchromes have a lower overall fecundity than infuscans females [24].

Infuscans females showed higher resistance to matings than both of the other morphs (Fig 3), but were found in copula with higher frequency and had a higher total mating rate (Fig 2). Stronger resistance towards mating attempts in infuscans females may result from more excessive male mating harassment in the field. If infuscans females are often clasped by males and subject to unwanted mating attempts, this may in turn select for higher female resistance post-clasping. Lower mating probabilities and mating rates of androchrome females may be either due to androchrome females avoiding male mating attempts by being male mimics or by behaving aggressively towards males [28], both of which could reduce unwanted male mating harassment. Thus, whereas males may have difficulties in visually detecting androchrome females, due to male mimicry, infuscans females may be easier to detect but once clasped, they are more resistant.

Whereas androchrome females appeared to be insensitive in terms of their fecundity to the number of matings (Fig 4a), infuscans females showed a more complex relationship with fecundity being maximized with either few or many matings (Fig. 4b). The non-linear relationship between fecundity and number of matings indicates disruptive selection on the number of matings in infuscans females, and that they would neither be expected to suffer from sperm-limitation at low male densities or fecundity costs from excessive multiple mating at high male densities. Alternatively, the non-linear pattern could be due to the quality differences between individuals, so that the infuscans females who have survived in the field in spite of many matings also have intrinsically higher vigour and fecundity. Our findings of a non-linear relationship for fecundity differ from the usual negative linear relationships between survival and number of matings for multiply mated females that have been documented in previous laboratory studies of insects [29]. Although non-linear (quadratic) relationships between the number of matings and fecundity have been suggested before [29], [30], the U-shaped shaped fecundity-relationship has, to our knowledge, never been documented in natural populations.

In conclusion, we have shown that the difference in mating strategy can lead to different fitness consequences in different morphs and can potentially maintain multiple morphs within populations. Sexual conflict over mating may not only involve visual traits like colouration differences between morphs [31] but could involve combinations of morphological and behavioural traits leading to different fitness consequences in different morphs. For instance, in the system we have described here, females could either avoid males by evolving visual traits like male mimicry (i. e. androchrome females) or by evolving strong behavioural resistance towards male mating attempts (i. e. infuscans females). Our study is one of very few examples of the fecundity consequences of multiple mating in the field. Female morphs in this system have apparently developed different ways to cope with male harassment, and these morph-differences can potentially explain rapid fluctuations in morph frequencies between years [15]. These data provide an empirical example of how sexual conflict can maintain multiple genetic and phenotypic clusters within populations rather than leading to speciation [11]. The female morphs in this system fluctuate rapidly in frequencies between years [24] due to frequency-dependent selection in favour of rare morphs [15]. Here we have shown that the different female morphs cope with male mating harassment in different ways. Conflict between males and females over the number of matings in this system can potentially explain the rapid morph-frequency fluctuations in this species.

## Materials and Methods

### Study species

The damselfly, *Ischnura elegans*, has a female limited polymorphism with females occurring in three discrete morphs, Androchromes (A), Infuscans (I) and Infuscans-obsoleta (IO). Androchrome females have both male-like colouration (blue) and male-like melanin patterning, and are considered to be “male mimics” (Fig 1a). Infuscans females have brownish to greenish colouration and differ from males in colour, although they have male-like melanin-patterning (Fig 1b). Infuscans-obsoleta females have pinkish to reddish colouration and have only weak melanin patterning (Fig 1c). Female morph development in *I.elegans* is controlled by a single locus with three alleles. These alleles form a dominancy hierarchy where A>I>IO[25] and where the six genotypes give rise to three visible phenotypes (Fig 1; For more information on the morphs see [24]). *I.elegans* is found throughout Europe with the northern end of its range occurring in Southern Sweden. During their reproductive season, males search out females, and once a female is located the male attempts to grab the females prothorax [32]. The males use claspers on the end of the male abdomen (cerci) and if the female is successfully grabbed they form what is known as the tandem position [32]. The female can then respond by bringing her abdomen up to join the males genitals forming a wheel so mating can take place [32]. Males have last male sperm precedence [33] and are non-territorial [32]. Females mate with multiple males [33].

### Field work and experiments

We collected and marked males from 3 different populations outside Lund in southern Sweden (Lomma (n = 1042), Höje Å 6 (n = 454), and Vombs Vattenverk (n = 374) during June and July of both 2005 (n = 725) and 2006 (n = 1215). Males were caught and marked with one out of five different colours of fluorescent dust [27]. Males were dusted in two separate places on their body; on the clasping organ located at the end of the abdomen (cerci) and on the genitalia. After dusting males were released. We returned to each population at regular intervals over the field seasons after each dusting session and caught as many single females and females found in copula. Morph frequency estimates were also estimated from captures of females caught without any dust. We returned to an indoor laboratory with caught females and checked for the presence of fluorescent dust under a dissecting microscope. We checked for the presence of fluorescent dust on the thorax, which indicates a male clasping attempt, and on the female's genitalia, which shows that mating had occurred with a dusted male. Females were subsequently set up in oviposition jars and left to lay eggs for two days before being released. After three days, the eggs were counted [24]. Although our fecundity estimate is only a component of the total female life-time fecundity, this fitness component may reflect 10–50% life-time fecundity in damselflies [32].

### Statistical analysis

All statistical tests were performed using Statistica [34]. We examined the differences between all the morphs caught in copula (1 = mated) and those caught singly (0 = non mated) across both years using a generalized linear models (GLZ) with binomial error structure and with a logit link function (Likelihood Type 3-test), controlling for year and population. The proportion of multiply mated females among all females with fluorescent dust with at least one mating (1 = 2 or more matings, 0 = 1 mating) were also analyzed using a similar GLZ-model. The average number of matings obtained by each morph was calculated from all females found with powder from one or several males, and the differences analysed using a general linear model (GLM) with year and population as random effects.

The relationship between the number of matings and the number of male clasping attempts was investigated with a GLM with number of matings as the dependent variable and population, year, morph, male clasping attempts, and the interaction term between morph and male clasping attempts as predictors. Population and year were added as random effects. This regression model did not include an intercept as it is not biologically possible for a female to mate without first being clasped by the male. We used the upper confidence limits of the regression slopes for each of the morphs to assess whether the regression lines were significantly different from the (hypothetical) 1∶1 slope, which reflects a situation in which every clasping attempt by a male ends in a mating, i.e. complete lack of female resistance.

The effect of multiple mating and male clasping attempts on fecundity was analyzed for only two of the morphs: Androchromes and Infuscans, due to the low sample size (n = 9) and few matings of the rarest morph (Infuscans-obsoleta). The fecundity data were poisson distributed so we used a GLZ-model with poisson error structure, including year and population as factors. The deviance parameter in the GLZ-model was rescaled to correct for over dispersion. Finally, morph-specific fitness functions (female fecundity) were visualised using Dolph Schluter's cubic splines program [35]. We selected the smoothing parameter (lambda) for the splines that minimized the General Cross Validation (GCV) score, in accordance with previous studies [35].
